# Cardiovascular Changes in Animal Models of Metabolic Syndrome

**DOI:** 10.1155/2013/761314

**Published:** 2013-03-14

**Authors:** Alexandre M. Lehnen, Bruno Rodrigues, Maria Cláudia Irigoyen, Kátia De Angelis, Beatriz D'Agord Schaan

**Affiliations:** ^1^Laboratório de Experimentação Animal e Laboratório de Cardiologia Celular e Molecular, Instituto de Cardiologia do Rio Grande do Sul/Fundação Universitária de Cardiologia do Rio Grande do Sul, Porto Alegre, Brazil; ^2^Divisão de Endocrinologia, Hospital de Clínicas de Porto Alegre, Universidade Federal do Rio Grande do Sul, Porto Alegre, Brazil; ^3^Laboratório do Movimento Humano, Universidade São Judas Tadeu, São Paulo, Brazil; ^4^Unidade de Hipertensão, Instituto do Coração (InCor), Faculdade de Medicina da Universidade de São Paulo, São Paulo, Brazil; ^5^Laboratório de Fisiologia Translacional, Universidade Nove de Julho, São Paulo, Brazil

## Abstract

Metabolic syndrome has been defined as a group of risk factors that directly contribute to the development of cardiovascular disease and/or type 2 diabetes. Insulin resistance seems to have a fundamental role in the genesis of this syndrome. Over the past years to the present day, basic and translational research has used small animal models to explore the pathophysiology of metabolic syndrome and to develop novel therapies that might slow the progression of this prevalent condition. In this paper we discuss the animal models used for the study of metabolic syndrome, with particular focus on cardiovascular changes, since they are the main cause of death associated with the condition in humans.

## 1. Introduction

According to the International Diabetes Federation [1], metabolic syndrome (MS) is clinically characterized by central obesity (waist circumference ≥94 cm for men and ≥80 cm for women), and at least two of these risk factors: high triglyceride levels (≥150 mg/dL); low HDL cholesterol (≤40mg/dL); high blood pressure levels (systolic blood pressure ≥130 mmHg and/or diastolic blood pressure ≥ 85 mmHg); and fasting plasma glucose levels ≥100 mg/dL. This cluster of cardiovascular risk factors is intrinsically related to increased incidence of diabetes mellitus [2] and cardiovascular mortality [3]. 

**Figure 1:**
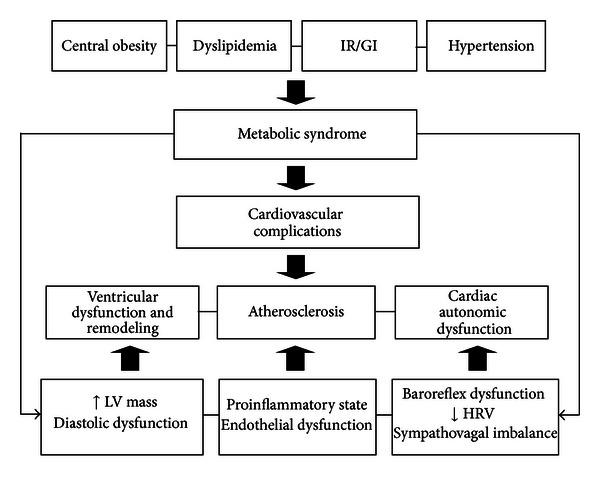
Components of the metabolic syndrome and its relation to the principal cardiovascular phenotypes. IR/GI: insulin resistance/glucose intolerance; HRV: heart rate variability; LV: left ventricular.

Studies have demonstrated several MS-induced abnormalities of cardiac geometry and function. Both increased left ventricular mass and relative wall thicknesses, as well as rapid deceleration time, have been found in hypertensive subjects with MS when compared with a hypertensive cohort without the syndrome [4]. On the other hand, patients with MS have presented left ventricular diastolic dysfunction independent of ventricular mass [5]. In addition, it is well known that insulin resistance plays a key role in MS and as such contributes to the development of premature cardiovascular atherosclerosis, independent of the association with diabetes and obesity [6–8]. It seems reasonable to assume that the association of several risk factors, as in the MS, favors an increased incidence of cardiovascular diseases and death risks in humans. The main cardiovascular complications observed in MS can be seen in Figure 1. Thus, a better understanding of the pathophysiological mechanisms of this syndrome becomes of paramount importance in clinical practice.

Studies on animal models can be relevant as they mimic the aspects of the human disease as the development and maintenance of MS characteristics, particularly obesity, type 2 diabetes, dyslipidemia, and hypertension. Experimental models of MS may be genetic, chemically induced, or diet induced. Thus, the aim of this paper is to both describe and discuss the animal models used for the study of MS. A special focus was given to cardiovascular changes which can be seen in Tables 1, 2, and 3.

### 1.1. Genetic Models

#### 1.1.1. *db/db* Mouse

The *db/db* mouse (BKS.Cgm+/+Leprdb/j) is a genetic model widely used as MS animal model, as it presents a leptin receptor mutation which causes hyperglycemia and insulin resistance [9]. The cardiovascular changes observed in *in vivo* studies indicate an increased vascular contractility [10]. However, the evaluation of these animals with 8-9 weeks of age did not lead to changes in blood pressure as compared to their wild controls [11]. As the animals age (14-15 weeks), blood pressure rises, together with autonomic neural changes. Noradrenergic responsiveness of the heart is reduced and indications of sympathetic denervation are observed [12]. 

The increase in blood pressure of these animals is associated with an increase in plasma angiotensin converting enzyme activity and angiotensin II levels [11]. Using spectral methods for autonomic function evaluation, no changes in heart rate variability, blood pressure variability, or baroreflex function are observed [11, 13]. In addition, *db/db* mice show impaired cardiac functional reserve capacity during maximal beta-adrenergic stimulation (with dobutamine), which is associated with unfavorable changes in cardiac energy metabolism [14].

#### 1.1.2. KKAy, *ob/ob*, and *db/db* Mice

KKAy is a congenital strain established by the transduction of the yellow obese gene (Ay) into the moderate hyperglycemic KK strain [15]. KKAy mice are obese and have high blood pressure levels, increased urinary excretion of catecholamines, and exacerbated responses to sympathetic blockade, suggesting a sympathetic role in the genesis of their hypertension [16, 17]. 

The *ob/ob *mice have a mutation in the *ob* gene resulting in leptin deficiency [18]. Short-term direct blood pressure measurements [19] suggested that the *ob/ob *mice are hypotensive with low sympathetic nerve activity [20]. However, when blood pressure is measured chronically, that is, data are collected for 5 s every 2 min and are averaged for the light cycle (7 AM–4 PM) and the dark cycle (7 PM–5 AM) with radiotelemetry (24 h full-time), *ob/ob* mice remain hypertensive during the light period [21]. Interestingly, during the dark cycle, *ob/ob* mice show no difference in blood pressure. On examination of the data over 24 h (the dark and light cycles combined), *ob/ob* mice are normotensive compared with control rats. With the use of radiotelemetry, blood pressure was measured 24 h/day, which obviously includes every activity performed by the animals. Thus, behavior and feeding may be especially relevant since the *ob/ob* animals have leptin deficiency and eat throughout the day and at night, leading to the hypertension observed during the day. Furthermore, these animals develop left ventricular hypertrophy with decreased cardiac function at 24 weeks of age [22] and cardiac fibrosis after 20 weeks of age [23]. 

Finally, the *db/db* (C57BL/KsJ-db/db) mice have inherited an autosomal recessive mutation in the leptin receptor gene present on chromosome 4 [9]. The metabolic alterations most frequently observed in this strain are hyperglycemia, hyperinsulinemia, hypertriglyceridemia, hypercholesterolemia, high levels of nonesterified fatty acids, and reduced HDL cholesterol [24]. In addition to these changes, both infiltration with inflammatory cells and fibrosis were observed in the heart after 12 weeks of age. These mice also show vascular endothelial dysfunction, although no blood pressure changes are observed [25]. We studied the cardiovascular and autonomic phenotype of male *db/db* mice and evaluated the role of angiotensin II AT(1) receptors. Radiotelemetry was used to monitor 24 h blood pressure in mice for 8 weeks. Although there were no changes in heart rate variability and spontaneous baroreflex sensitivity between control and *db/db* mice, the results indicate an age-related increase in mean arterial pressure in *db/db* mice, which can be reduced by the antagonism of angiotensin II AT(1) receptors [11].

#### 1.1.3. Wistar Ottawa Karlsburg W (WOKW)

Wistar Ottawa Karlsburg W (WOKW) rats were developed from a Wistar rat outbred strain of the BioBreeding Laboratories. As observed in humans, the features of MS in this model depend on polygenic factors and may be due to a single-gene mutation [26]. WOKW presents hyperphagia, which leads to obesity. In addition to obesity, this animal model shows other metabolic alterations, such as dyslipidemia, hyperinsulinemia, and impaired glucose tolerance. Specifically, insulin resistance in this animal model may be linked to a mutation on chromosome 3 [26, 27]. Genomic scan studies have revealed a linkage of the MS and/or diabetes to a region on chromosome 3 (3q26-27), where the gene encoding adiponectin, apM1, is located [28]. One important clinical characteristic displayed by these animals was the impaired coronary function, due to increased alpha(1)-adrenoceptor-mediated coronary constriction (at 3 and 10 months of age), and to a seriously blunted beta-adrenoceptor-mediated coronary relaxation (at 16 months of age) [29].

#### 1.1.4. Zucker Obese Rats (*fa/fa*)

Zucker obese (ZO) rat model develops MS characterized by obesity since they are polyphagic due to a mutation in the leptin receptor [30, 31], insulin resistance, hypertriglyceridemia, and hypertension [32]. Obesity leads to an inflammatory state which is linked to reduced insulin sensitivity and expression of GLUT4 in adipose tissue, skeletal muscle, and heart [33, 34]. This is likely to be related to increased circulating free fatty acids which compete with glucose as energy substrate [35] and also inhibit the translocation of GLUT4 to the cell membrane [36].

Myocardial fatty acid uptake and utilization lend support to the hypothesis that myocardial insulin resistance is associated with cardiac dysfunction, characterized by increased left ventricle mass, reduction systolic function, and survival. Increasing fatty acid as fuel energy can lead to increased reactive oxygen species, thus contributing to structural and functional damage in the myocardium [37].

Accordingly, 9-week-old ZO rats show diastolic dysfunction with preserved ejection fraction [33]. These abnormalities occur prior to the onset of hypertension, which becomes elevated above control levels around 12 weeks of age [34]. Furthermore, cardiovascular complications similar to human obesity also include higher resting sympathetic nerve activity and reduced heart rate variability [35, 36] which were observed in ZO rats—attenuated baroreflex-mediated changes in sympathetic nerve activity to vascular targets [32]. These are due to impairments in sympathetic and parasympathetic control of the heart [37, 38].

#### 1.1.5. Zucker Diabetic Fatty Rats (*fa/fa*)

Zucker Diabetic Fatty Rats (ZDF) phenotype originated from selective breeding of Zucker rats with high glucose levels, which developed diabetes after 10 weeks of age. ZDF presents hyperphagia, as a result of a nonfunctioning leptin receptor, which in turn leads to obesity similar to the prediabetic state in humans [39]. Furthermore, hyperglycemia in the ZDF model is different from that observed in the ZO; ZDF rats do not have sufficient pancreatic *β*-cell function. 

It has been observed that changes in left ventricular chamber morphology occurred in the untreated ZDF animals as early as 16 weeks of age [40]. Using positron emission tomography and echocardiography, van den Brom et al. (2009) found an increase in myocardial fatty acid oxidation, a reduction in insulin-mediated myocardial glucose utilization, associated with impairment of myocardial function in ZDF rats with 14 weeks of age [41]. In addition, vascular and neural dysfunction observed in ZDF rats with 12 weeks of age has been improved with vasopeptidase inhibitors [42].

In addition, this animal model shows an increase in plasma angiotensin converting enzyme activity and angiotensin II levels that, associated with high glucose levels, can lead to advanced nephropathy. This may be the reason by which ZDF rats started to die at 50 weeks of age (63 weeks for the control group), in association with an abrupt increase in blood urea nitrogen, suggesting that the cause of death was renal insufficiency [43].

#### 1.1.6. DahlS.Z-Lepr^fa^/Lepr^fa^ Rat

One of the most recently created MS models is the DahlS.Z-Lepr^fa^/Lepr^fa^ rat (DS/obese). This MS model was established by crossing Dahl Salt rats and Zucker rats with the missense mutation in the leptin receptor gene (Lepr). The animals, once fed with a normal diet, developed obesity, as well as hypertension, dyslipidemia, insulin resistance, and type 2 diabetes. In addition, these animals developed cardiac hypertrophy, as well as renal and liver damage, which may account for their premature death. Hattori et al. (2011) showed that in an experimental period of 18 weeks, 13 (65%) of 20 DS/obese rats died (seven from renal failure, two from cerebrovascular events, and four from sudden cardiac death); there were no deaths in the control group [44]. In addition, Murase et al. (2012) have recently demonstrated that body weight, as well as visceral and subcutaneous fat mass, was significantly increased in DS/obese female rats, which was associated with diastolic dysfunction and marked left ventricle hypertrophy and fibrosis [45]. Myocardial oxidative stress and inflammation, serum insulin, and triglyceride were also increased in DS/obese rats compared with DS/lean rats.

#### 1.1.7. Otsuka Long-Evans Tokushima Fatty Rats

Otsuka Long-Evans Tokushima Fatty (OLETF) rats are a cholecystokinin 1 receptor knockout model which become obese secondarily to hyperphagia [46]. The increased food intake is characterized by a large increase in meal size with a decrease in meal frequency which is not sufficient to compensate for the meal size increase. As a result, these animals usually present hyperglycemia after 18 weeks of age, mild obesity, and diabetes mellitus, more frequently observed in males [47]. However, insulin resistance in OLETF rats emerges at 12 weeks of age, before the impairment of pancreatic *β*-cell function [48]. 

Mizushige et al. (2000) have previously demonstrated that OLETF rats present diastolic dysfunction in their prediabetic state (15 weeks), observed by a prolongation in deceleration time and a decrease in amplitude of peak velocity of the early diastolic filling wave, without changes in the blood pressure and heart rate. In addition, the researchers observed extracellular fibrosis and abundant transforming growth factor-*β*1 receptor II in the left ventricle of these rats [49]. While still in the prediabetic stage, OLETF rats exhibited a lower coronary flow reserve and increased coronary vascular resistance during hyperemia, which was associated with increased wall-to-lumen ratio and perivascular fibrosis [50]. In the late stage of diabetes (22 and 62 weeks of age), it was demonstrated that these animals displayed an impairment in diastolic function and changes in the geometry of conductance and resistance arteries [51]. 

#### 1.1.8. Goto-Kakizaki Rats

The Goto-Kakizaki (GK) model was created by selective breeding of an outbred colony of Wistar rats, selected for high glucose levels in an oral glucose tolerance test [52]. These animals develop hyperglycemia after 4 weeks of age [53] and increased liver and plasma lipid concentration after 8 weeks of age [54].

After 16 weeks of age, at prediabetic state, GK rats displayed left ventricle remodeling with marked hypertrophy of cardiomyocytes and increased extracellular matrix deposition, culminating in increased heart size [55]. Despite the progressively worsening of glucose metabolism derangement, at 18 months of age the contractile function of the heart appears to be well preserved, as observed by maintenance of amplitude of shortening in electrically stimulated myocytes [56]. In addition, untreated GK rats presented mild hypertension and a blunted vascular relaxation by acetylcholine and sodium nitroprusside when compared to control animals [57]. 

### 1.2. Chemically Induced Models

#### 1.2.1. Monosodium Glutamate-Induced Spontaneously Hypertensive Rat

We studied in our laboratory the cardiovascular autonomic function of obesity induced by monosodium glutamate (MSG) in a normotensive model. Male Wistar rats receiving MSG neonatal treatment showed metabolic abnormalities, as well as increased body weight, Lee index, epididymal white adipose tissue, and insulin resistance. In addition, these animals exhibited reduced glucose/insulin index (−62.5%), and increased insulin secretion during glucose overload (39.3%), and hyperinsulinemia. These rats showed a slight increase in mean arterial pressure with no difference in the heart rate from their controls. However, they showed cardiovascular autonomic dysfunction, as shown by reduced baroreflex sensitivity and vagal and sympathetic effects when compared to their controls. We observed a reduced sympathetic effect which was not followed by changes in the basal heart rate or tachycardic responses to arterial pressure changes [58].

Recently, we carried out studies on cardiovascular abnormalities of the spontaneously hypertensive rat (SHR) [59, 60] treated with MSG in the neonatal period [61]. Although this animal model is not new [62, 63], no research, to our knowledge, has been undertaken using metabolic and cardiovascular parameters over time (3, 6, and 9 months of age). The use of MSG in genetically hypertensive rats led these animals to progressively increase body adiposity and triglyceride levels. Besides developing and maintaining insulin resistance, they presented low HDL cholesterol and increased inflammation state (high C-reactive protein, interleukin 6, tumor necrosis factor-*α* levels, and low adiponectin levels), which reached highest levels at 6 and 9 months of age [61].

In MS, hypertension is commonly associated with metabolic changes of the syndrome. Therefore, the use of a genetically hypertensive rats associated with MSG treatment, which leads to metabolic changes including obesity, seems to work well as an MS model resembling the human disease. However, early studies showed that SHR treated with MSG did not maintain hypertension [62], but it should be noted that indirect tail-cuff methods were used. Thus, we evaluated blood pressure by direct method (analyzed on a beat-to-beat basis) at 3, 6, and 9 months in MSG-treated SHR compared to SHR and normotensive rats [61]. It was observed that mean blood pressure was similarly higher in SHR and MSG-treated SHR at all ages, when compared to normotensive rats. Furthermore, there were no changes in blood pressure over time in the groups studied.

The increased cardiovascular mortality which characterizes MS may be partially attributed to cardiac sympatho-vagal imbalance [64, 65]. Thus, we evaluated temporal cardiovascular autonomic dysfunction in this model by spectral analysis and observed that the changes in cardiovascular autonomic control evaluated up to 9 months are similar to those observed in hypertensive rats. At 9 months the animals maintained the hypertensive state, a feature of SHR, and presented reduced heart rate variability, decreased spontaneous baroreflex sensibility, and increased cardiac sympathovagal balance. In addition to these changes, it was observed that SHR and SHR with MSG-induced obesity showed increments of 6- to 8-fold of systolic arterial pressure variability at 6 and 9 months, as compared to normotensive rats. Interestingly, at 6 months, MSG-induced SHR had increased systolic arterial pressure variability when compared to only SHR. Taken together, SHRs with MSG-induced obesity show an impairment of the cardiovascular nervous control. Sympathetic activation plays an important role in the pathogenesis of insulin resistance [66–68] and in the activation of the renin-angiotensin system [69], which are related to cardiovascular autonomic control dysfunction of MS [64]. 

#### 1.2.2. Streptozotocin Administration

The injection of streptozotocin (STZ) to mice, rats, and rabbits has been widely used as a model of type 1 diabetes. Streptozotocin destroys pancreatic *β* cells, resulting in a diabetic syndrome in animals, characterized by hyperglycemia, hypoinsulinemia, glycosuria, and body weight loss [70, 71]. These rats have systolic and diastolic dysfunction at rest, as evaluated by left ventricular catheterization and echocardiography, as well as reduced capacity for cardiac adjustment to volume overload and maximal oxygen consumption [72, 73]. Furthermore, STZ induces reduction in baroreflex-mediated bradycardia and tachycardia, as well as impairment in cardiac vagal tone in the face of unaltered sympathetic tone [71, 74, 75].

Although well accepted in the literature as a model for type 1 diabetes, the injection of STZ alone does not allow it to mimic the characteristics of MS in humans, since these animals show hypoinsulinemia, loss or no change in body weight, and hypotension [71, 74, 75]. An alternative approach, previously used, is the administration of low doses of STZ (25 mg/Kg) associated with high fructose diet in rats. This combination induced mild hyperglycemia and hypertriglyceridemia associated with mild fasting hyperinsulinemia and whole body insulin resistance, without significant increase in body weight [76]. In addition, the authors also observed decreased left ventricular contractile function and reduced myocardial metabolic efficiency.

### 1.3. Diet-Induced Models

#### 1.3.1. Fructose Overload

Since 1978 the mean daily intake of added fructose and total fructose has increased in both sexes and all age groups, having as the main sources of consumption soft drinks and other sweetened beverages [77, 78]. Fructose overload in drinking water or chow has been used to promote metabolic, hemodynamic, structural, and functional derangements in rodents. Fructose overload in experimental animals has been associated with high triglyceride levels, adiposity, insulin resistance, and glucose intolerance [79–86]. These animals have been used by our group in order to understand the various aspects of obesity, dyslipidemia, and insulin resistance-associated cardiovascular changes [81–88]. We observed that an increased sympathetic modulation to the vessels and heart preceded metabolic dysfunction in fructose-consuming mice, thus suggesting that changes in autonomic modulation may be a triggering mechanism underlying the cluster of symptoms associated with cardiometabolic disease [83–86, 88].

Animal studies have shown strong associations between high fructose intake and the onset of arterial hypertension. According to Farah et al., mice which received a high fructose diet showed higher blood pressure and heart rate in the dark (active) period when compared with the light (resting) period [87]. This augment in blood pressure was related to an increase in the blood pressure variability and, consequently, to increased vascular sympathetic modulation. This is a relevant finding since during the dark period the mice are active (grooming, eating, and drinking) and the sympathetic activity should be high. On the other hand, it was displayed that female fructose-fed rats presented reduced vagal tonus, unaltered sympathetic tonus, and intrinsic heart rate [81]. However, this study also showed that although the sympathetic tonus remained unchanged, the autonomic balance (sympathetic/parasympathetic) was altered, leading to a sympathetic predominance. A positive correlation between reduced cardiac vagal tonus and insulin resistance was also found, thus reinforcing the relationship between autonomic and metabolic dysfunction [81]. 

Since arterial baroreflex influences both sympathetic and parasympathetic outflow, disorders in autonomic neuronal pathways (efferent or afferent) may affect cardiovascular health and be related to high blood pressure. High fructose consumption induces an impairment of baroreflex sensitivity, as evaluated by heart rate responses associated with arterial pressure changes induced by vasoactive drugs [81, 86], as well as by linear regression and alpha-index [84]. Furthermore, unpublished data from our laboratory demonstrated that impairment of baroreflex sensitivity was positively correlated with the number of elastic lamellae in the ascendant aorta. This data suggest that the possible loss of distensibility of the aorta may be associated with changes in baroreflex sensitivity, since the mechanical stress of the arterial wall cannot be effective to properly trigger the mechanoreceptors.

Regarding the rennin-angiotensin system, it has been widely acknowledged that high-fructose diet induces increased angiotensin II plasma levels, which contributes to hypertension, insulin resistance, and dyslipidemia [89], and may account for cardiac remodeling [80, 90] and vascular oxidative stress [91] in this MS model. In fact, it appears that angiotensin II promotes fibroblast proliferation due to activation of the angiotensin II type 1 receptor, which results in increased collagen type III expression and accumulation in the heart [92]. Also, there is evidence that the insulin-resistant state is associated with angiotensin II type 1 receptor upregulation and increased endothelial (aorta) superoxide anion levels, which is likely to be caused by an increase in NAD(P)H oxidase expression in fructose-fed rats [91]. Furthermore, it should be stressed that angiotensin 1a receptors are critical in mediating the response to a high-fructose diet and the resultant state of glucose intolerance, because in the absence of these receptors, a fructose diet decreases the blood pressure, as observed in angiotensin 1a knockout mice [87]. 

As an important consequence of these metabolic, hemodynamic, autonomic, and structural changes displayed in this MS model, our group recently demonstrated that fructose overload promoted changes in left ventricular morphometry, diastolic dysfunction, and increased cardiac effort, as evidenced by the increase in the myocardial performance index [85]. Other researchers have found similar results for Wistar rats receiving a 10% fructose overload for 8 weeks [93].

#### 1.3.2. Sucrose Overload

Sucrose is a disaccharide composed by one molecule of glucose linked to one molecule of fructose through an *α* 1–4 glycoside bond [94]. Similar to fructose, sucrose induces MS in animals, as increased plasma concentrations of insulin, leptin, triglycerides, glucose, and free fatty acids and impaired glucose tolerance were shown [95]. Sucrose-treated rats (32% in drinking water) revealed early abnormalities in diastolic function (2.5 weeks of treatment) followed by late systolic dysfunction and concurrent alterations in myocardial structure (10 weeks of treatment). Furthermore, the authors demonstrated that after 10 weeks of sucrose treatment the animals presented reduced calcium uptake in sarcoplasmic reticulum of cardiomyocytes [96].

In addition, animals treated with an 8% sucrose solution developed hypertension and tachycardia after 2 weeks of treatment, and this was not related to weight gain. The authors concluded that sucrose ingestion may stimulate the ventromedial hypothalamus to increase sympathetic activity and elevate blood pressure in rats [97]. 

#### 1.3.3. High Fat Diet

High fat diets have been used with fat fractions between 20% and 60% energy as fat, and the basic fat component varies between animal-derived fats and plant oils, for example, corn, coconut, or safflower oil [98]. After 10 weeks of a high fat diet, rats displayed high fat mass, insulin resistance, and hyperleptinemia, typically associated with obesity [99]. Rabbits receiving a high fat diet during 3 weeks presented an impairment in leptin sensibility, along with increased mean arterial pressure, heart rate, and plasma norepinephrine concentration. Renal sympathetic nerve activity was also higher in high fat diet rabbits when compared to control diet rabbits and was correlated to plasma leptin [100]. Furthermore, Fardin et al. (2012) have demonstrated that the baroreceptor dysfunction which controls renal sympathetic nerve activity is an initial change in the obesity-induced high fat-fed rats, which might be a predictor of sympathoexcitation and hypertension associated with obesity [101].

The renin-angiotensin system has been involved in the hypertension genesis linked to obesity [102]. In fact, Boustany et al. (2004) observed that both angiotensinogen gene expressions in retroperitoneal fat mass and plasma angiotensinogen concentration were increased in rats receiving high fat diet, thus showing increased activity of the adipose and systemic rennin-angiotensin system in obesity-related hypertension [103]. 

#### 1.3.4. Cafeteria Diet

Proponents of chemically induced MS models have been challenged by researchers who argue for diet-induced models. The latter contend that these reflect more accurately the condition of human obesity when compared to chemically induced or genetic modifications [104].

Thus, several diet-induced experimental models have been proposed, suggesting that there is an aggregation of factors in MS. The cafeteria diet model [105] is a fine example of a diet-induced experimental model. In this model, animals are allowed free access to standard chow and water while concurrently given highly palatable, energy dense, unhealthy human foods *ad libitum*, which promotes voluntary hyperphagia. This diet results in weight gain, increased fat mass, glucose intolerance, and insulin resistance [106, 107].

Wistar rats fed a cafeteria diet showed, in addition to the classical metabolic disorder, cardiovascular alterations, such as increased heart rate and blood pressure [108]. Cafeteria diet has been shown to lead to impairment of the endothelium-derived hyperpolarization mechanism, in particular, potassium channel signaling mechanisms [109]. Thus, this finding may explain, at least in part, the link between cafeteria diet and increased blood pressure in rats; this link is further reinforced by understanding that vascular tone refers to the balance between constrictor and dilator actions and influences the control of blood flow and pressure, and this phenomenon is influenced by endothelium-derived hyperpolarization mechanism.

Although normotensive rats fed a cafeteria diet showed an increase in blood pressure, this diet may have a better outcome if used in SHR, since this animal model has cardiac disorders, for example, increased sympathetic activity, hypertension, and cardiac hypertrophy [59, 60] which may be potentiated by the cafeteria diet. Spontaneously hypertensive rats fed with cafeteria diet for 12 weeks showed metabolic changes similar to those of the MS, for example, high plasma levels of glycemia, insulin, triglyceride, leptin, and obesity. Furthermore, animals maintained hypertension, a common feature of SHR [110]. Many studies have dealt with the autonomic dysfunction of SHR by spectral analysis [111, 112].

## 2. Conclusions

There is considerable evidence to support the hypothesis that the cluster of complications from metabolic syndrome status converges to a derangement of the cardiovascular system. In fact, cardiovascular diseases are the leading cause of morbidity and mortality in this condition. The use of different experimental models that mimic the metabolic derangements seen in this syndrome is extremely important in order to better understand the mechanisms involved in cardiovascular changes caused by metabolic syndrome. New approaches, such as genetically modified models, can provide mechanistic information on the genetic and environmental factors involved in the development of cardiovascular dysfunction in the metabolic syndrome.

## Figures and Tables

**Table 1:** Main cardiovascular findings in genetic models of metabolic syndrome.

Models	Cardiovascular changes
*db/db* mouse	(i) Increased vascular contractility
	(ii) High blood pressure levels (14-15 weeks of age)
	(iii) Sympathetic denervation (14-15 weeks of age)

KKAy mice	(i) High blood pressure levels
	(ii) Sympathetic alterations

*ob/ob* mice	(i) Hypotensive with low sympathetic nerve activity
	(ii) Cardiac fibrosis (20 weeks of age)
	(iii) Left ventricular hypertrophy (24 weeks of age)
	(iv) Decreased cardiac function (24 weeks of age)

*db/db* mice	(i) Vascular endothelial dysfunction
	(ii) No or low blood pressure changes
	(iii) No changes in heart rate variability
	(iv) No changes in spontaneous baroreflex sensitivity

Wistar Ottawa Karlsburg W	(i) Impaired coronary function
	(ii) Increased alpha(1)-adrenoceptor-mediated coronary constriction (3 and 10 months of age)
	(iii) Seriously blunted beta-adrenoceptor-mediated coronary relaxation (16 months of age)

Zucker obese rats	(i) Diastolic dysfunction with preserved ejection fraction (9 weeks of age)
	(ii) High blood pressure levels (12 weeks of age)
	(iii) High resting sympathetic nerve activity
	(iv) Reduced heart rate variability

Zucker Diabetic Fatty	(i) Increased myocardial fatty acid oxidation
	(ii) Reduction of insulin-mediated myocardial glucose utilization (14 weeks of age)
	(iii) Reduction of left ventricular chamber

DahlS.Z-Lepr^fa^/Lepr^fa^	(i) Diastolic dysfunction
	(ii) Marked left ventricle hypertrophy and fibrosis
	(iii) Myocardial oxidative stress

Otsuka Long-Evans Tokushima Fatty	(i) Diastolic dysfunction (15 weeks)
	(ii) No changes in the blood pressure and heart rate
	(iii) Extracellular fibrosis and abundant transforming growth factor-*β*1 receptor II in the left ventricle
	(iv) Low coronary flow reserve
	(v) Increased coronary vascular resistance

Goto-Kakizaki	(i) Left ventricle remodeling with marked hypertrophy
	(ii) Increased extracellular matrix deposition
	(iii) Mild hypertension
	(iv) Blunted vascular relaxation by acetylcholine and sodium nitroprusside

**Table 2:** Main cardiovascular findings in chemically induced animal models of metabolic syndrome.

Models	Cardiovascular changes
MSG-induced SHR	(i) High blood pressure levels
	(ii) Reduced heart rate variability
	(iii) Decreased spontaneous baroreflex sensibility
	(iv) Increased cardiac sympathovagal balance
	(v) Increased systolic arterial pressure variability

Streptozotocin administration	(i) Systolic and diastolic dysfunction at rest
	(ii) Reduced capacity for cardiac adjustment to volume overload
	(iii) Reduction in baroreflex-mediated bradycardia and tachycardia
	(iv) Impairment in cardiac vagal tone

MSG: monosodium glutamate; SHR: spontaneously hypertensive rat.

**Table 3:** Main cardiovascular findings in diet-induced animal models of metabolic syndrome.

Models	Cardiovascular changes
Fructose overload	(i) High blood pressure levels
	(ii) High heart rate
	(iii) Increased blood pressure variability
	(iv) Increased sympathetic modulation to the vessels and heart
	(v) Vascular oxidative stress
	(vi) Changes in left ventricular morphometry
	(vii) Diastolic dysfunction
	(viii) Increased cardiac effort

Sucrose overload	(i) High blood pressure levels
	(ii) High heart rate
	(iii) Diastolic function
	(iv) Systolic dysfunction
	(v) Alterations in myocardial structure due to reduction of calcium uptake in sarcoplasmic reticulum of cardiomyocytes

High fat diet	(i) High blood pressure levels
	(ii) Baroreceptor dysfunction that controls the renal sympathetic nerve activity

Cafeteria diet	(i) High blood pressure levels
	(ii) High heart rate
	(iii) Impairment of the endothelium-derived hyperpolarization mechanism
	(iv) Autonomic dysfunction
